# On Hospital Construction

**Published:** 1887-11-12

**Authors:** Keith D. Young


### On Hospital Construction.

By Keith D. Young, F.R.I.B.A.

*Read at the Meeting of the Hospitals Association, Nov. 9th, 1887.*

I propose to confine myself to two or three points only which seem to offer scope for fruitful discussion, or which have some special interest in view of recent developments.

The first point is that of the general arrangement or plan-planning of a hospital, with particular reference to the relation between one ward and another, and between the wards and the administrative parts of a hospital.

The importance of a free air space round wards was recognised as long ago as 1788, by Mons. Tenon, whose model plan for a hospital shows a complete separation between the wards and the administrative offices, and is in many other respects greatly in advance of anything that had gone before, and much that followed after. It was not until well within the present century that what is known as the pavilion system became an established fact. One of the earliest, if not the earliest, hospital of the pavilion type was the Lariboisiere, in Paris, which was finished in the year 1854. Our own St. Thomas's Hospital was opened in 1871, and since then many other hospitals of varying size have been built upon the same system. The chief feature in the pavilion type of hospital is that the enclosing walls of the wards are entirely free and open to the air throughout their whole length, and that by this means it is possible to obtain on each side of the ward an equal number of windows immediately facing each other. Thus cross ventilation to its utmost extent is secured for the whole length of the ward.

The points that I wish to draw particular attention to are the corridors connecting the ward pavilions with each other, and the administration, and the position of the staircases. At the Lariboisiere these corridors are one storey in height only, but are closed in on each side. At St. Thomas's they are two storeys, and also are closed in. At the new Hotel Dieu they are two storeys high, the upper storey being open at the sides, the lower closed. At the Royal Infirmary, Edinburgh, the surgical pavilions are connected by a two storey, closed-in corridor; the medical pavilions by a one storey similar corridor. In all the hospitals just referred to the staircases, and in most cases the lifts, are so placed as to be in direct atmospheric communication with the ward, and, as a consequence, to form shafts of air connection between one ward and all the others. The closed-in corridors complete the chain of connection by which all the wards are placed in direct aerial communication with each other and with the administrative offices. It would seem, therefore, that the question of the aerial separation between the wards and the administrative offices, and between one ward and another, is not satisfactorily solved in these earlier instance of the pavilion type of hospital.

If we turn for a moment to some recent foreign hospitals, we shall find that this question has been dealt with in a way that has not as yet been attempted in this country.

In the Hospital of St. Denis, near Paris, and the University Hospital at Heidelberg, we have examples of the most complete disconnection between the wards and the administrative offices, and also between the wards themselves, and in each case it will be observed that surgical cases are more carefully separated and sub-divided than medical cases. In the case of the St. Denis Hospital we have not only the ordinary medical and surgical cases which are received into the wards of general hospitals, but we have also two blocks of four single-bed wards each, for patients suffering from infectious fevers, so that (excluding mental diseases) this hospital is destined to deal with disease and injury in every conceivable form.

In both the hospitals just referred to the wards are raised above the ground on piers, and there is a free air space under the floors of 7·0 in height, the ground being sloped down at each side; so that not only is there the greatest possible air separation between each block, but the air currents have full play both around and beneath the buildings. By this means the ward floor itself is raised above all possibility of contamination by ground air, and the possibility of stagnation of air in the angle formed by the upright walls and the ground is entirely avoided.

Another example of the isolated pavilion plan is to be seen in the great Friedrichshain, or General Town Hospital, at Berlin, where the only means of communication between the different ward blocks are paved footways.

Two questions seem to me to arise out of the consideration of these hospitals: (1) Is there any advantage at all commensurate to the great increase of cost entailed in limiting the ward pavilions to one storey in height? and (2) Is it desirable or practicable to dispense altogether with closed-in corridors of communication, or even, as at St. Denis, to abolish covered ways entirely?

As to the first question, I must confess that for my own part I have been quite unable to discover any adequate reason for the limitation of wards to one storey only. In M. Tollet's system it becomes a necessity, as it is of the essence of his plan that the pointed arch form of roof should be preserved, and that the outlet ventilator should be at the apex of the arch. But given ample space around the wards in proportion to their height and the means of entirely separating the ward air of one floor from that of the floor below, I cannot see any advantage to be gained that would outweigh the enormous increase in cost which would be involved in places where, as in London, the value of land is calculated by the square inch. It has never, I think, been contended that the air from one ward could pass by way of the windows into the wards above; it is by way of staircases, passages, lifts, and shafts, and possibly by way of the floors when constructed in the ordinary way, that the circulation of air takes place. If, therefore, we so plan our wards that each is atmospherically distinct from the others, we shall, I think, attain the desired object almost as completely as if we adopt the principle carried out in Germany and France. As an illustration of the arrangement I have endeavoured to describe let me ask your attention for a moment to a hospital now being erected in London, and for the design of which I am, jointly with Mr. Henry Hall, responsible.

The plan represents the Great Northern Central Hospital at Holloway as it is intended to be. At present only the rectangular ward block, the back wing of the administration block, and the out-patient department and mortuary are in course of erection. The hospital consists really of six distinct buildings, all of which are or can be made absolutely distinct one from the other. The front block contains the board-room and offices, residence for officers, private wards for paying patients, and nurses' dormitories. The wing at the back contains the stores in the basement, surgery and consultation-room, nurses dining and sitting rooms, operating theatre with adjuncts, nurses' bed-rooms, and the kitchen offices on the top floor. In this wing too, are placed the main staircase and all the lifts.

To right and left of this central block, corridors lead to the two ward blocks. These corridors are cross ventilated, and are shut off by doors from the ward blocks.

Personally, I should like to see the windows abolished and the corridors converted into simple covered ways; but in that case it would, I imagine, be necessary to provide means of closing in the sides in such a winter as last.

The ward blocks then, with their accessories, are practically quite as distinct from each other and from the main building as if they were in separate hospitals.

So far for horizontal separation. Vertical communication between one ward and those above is guarded against by the avoidance of any vertical shaft within the ward blocks, and by the construction of the floors, which are of solid concrete and iron.

The remaining two blocks are respectively the out-patient department and the mortuary, with post-mortem-room, etc.

To return to our second question, as to the necessity for covered communication between the various blocks in a hospital. There is much to be said on both sides of this question. On behalf of the absolute separation theory there is of course the perfect isolation of each building to be urged. The hospital becomes a series of houses, and from the point of view of the doctor who, in the course of his professional rounds visits many houses, there can be nothing objectionable in this. But it is with the kitchen arrangements that the most serious objection lies to the absence of covered ways, and one can readily imagine the inconvenience of having to wheel the food trolleys containing hot diets for the patients through a snowstorm. On the whole, I fancy the balance of opinion would be in favour of covered ways; these, if made sufficiently broad and low will afford ample protection from the weather. An example of this arrangement is to be seen at the South-Eastern Hospital of the Metropolitan Asylums Board, where it has, I believe, worked exceedingly well.

In discussing the question of the vertical separation between one ward and those above it, I referred to the construction of the flooring as being a point of importance in this respect.

The ordinary way of forming a floor is to place beams of timber or joists across from wall to wall, and to fix on their upper sides the floor boards, and below them the ceiling of lath and plaster. By this arrangement it will readily be seen that there is a space intervening between the ceiling and the floor boards. When, in a house that has been occupied for some time, the floor boards are taken up, it will be found that the upper surface of the ceiling below, between the joists, is covered with dirt and dust of all kinds. Now in a hospital ward dust is more emphatically "matter in the wrong place" than it is anywhere else. Dirt means disease, and disease in a surgical ward means all those forms of septic poison which are amongst the greatest enemies surgeons have to contend with.

When, therefore, the only barrier between one ward and another is a thin sheet of porous plaster and deal boards, which, if they fit ever so closely, still leave room for the passage of dust-laden air, it is impossible to say that the patients in the upper ward are safeguarded from emanations from the ward below. It is therefore absolutely necessary that a ward floor should be solid, and the best way to obtain this result is by forming the framework of the floor with iron beams and filling in the spaces with solid concrete. The wood floor—for in this country I do not think a cement or marble floor would be tolerated for a moment—is then laid down on the concrete without the intervention of any joists or beams whatever. A difficulty necessarily inherent in any form of wood floor is that of joists. Wherever joists exist there will be a crevice, and be the crevice ever so small it is a lurking-place for the minute organisms with which the air is laden. The best mode of obviating this difficulty seems to be, first, to have the floor made of the hardest, most close-grained wood obtainable, such as teak or oak; secondly, to secure that the wood itself, whatever is used, is as absolutely dry and well-seasoned as it is possible to make it; and, thirdly, to treat the surface either by wax polishing, or, better still, by the paraffin process. This latter consists in forcing paraffin into the pores of the wood by hot irons, and then polishing the whole surface with a solution of paraffin. A floor thus treated becomes absolutely impervious. A wood called Jarral, one of the Eucalyptus family, has been suggested for ward floors. It is about the same cost as oak, and appears to be well adapted for the purpose. Whether it has the antiseptic properties claimed for it I am unable to say.

Before leaving the subject of the wards there are two or three points upon which I would say a word: First, with regard to those narrow windows that are to be seen at each of the four corners of the rectangular wards of the Great Northern Hospital. It has been pointed out by many observers that the cases in the beds at the extreme ends of wards have persistently fared worse than those in the other beds; and the reason that most obviously suggests itself is that in the angles the ventilation is apt to be more sluggish than in the central parts—that the air has, in fact, a tendency to become sluggish. To overcome this difficulty we have added slightly to the space occupied by the end beds, and have placed those narrow windows shown on the plan between each end bed and the return wall. Another point I wish to refer to is that of angles. The rounding of the internal angles in a ward is no new thing. In the horizontal angles it is done to prevent the accumulation of dust in corners—everyone knows how dust will accumulate in nooks and corners, and how impossible it is to sweep well into an angle. In the vertical angles and the angles made by the ceiling and the wall the object is to promote ventilation, the fact being that there is a tendency to stagnation of air in these parts. The effect of this is readily seen in a room where the walls and ceiling have become discoloured by time. In all the inner angles will be seen a white line showing itself conspicuously by contrast with the surrounding dirt. The cause of this is imperfect circulation of air, and therefore of the dust borne on the air. In a modern hospital at Molenbeck, St. Jean, near Brussels, the internal angles of the walls are rounded to a radius of about six feet. I would, however, go further than this and convert into a segment every angle that can by any possibility be altered. The corners of door panels, of the panes in a window, the angles in the ward furniture—in short, everywhere an angle exists that can possibly be rounded, rounded it should be made.

A certain number of patients, more or less ill, are for convenience of nursing placed together in one large ward. It is necessary to supply to each of those patients such an amount of fresh air as will keep the atmosphere constantly diluted to a certain given standard of purity. And this must be done without draught, and must, moreover, be entirely under the control of those in charge of the ward. In addition to this, it is necessary to provide means of warming the wards to a suitable temperature in cold weather. The natural system is, as I have said, dependent on windows or other openings in the external walls, aided at times by the smoke flues from the fireplaces. By these means the great majority of English hospitals are ventilated, and it has been held by very eminent authorities, that for our climate nothing more suitable can be devised. The chief objection to this system is, that wards readily become draughty, and therefore much mischief ensues, especially in cases of lung disease. I venture to think that with care this difficulty can be overcome. The special form of window to be used is an important factor in this. Formerly, the ordinary double-hung sliding sash was pretty generally adopted; as usually made it admits air exceedingly well, but in a perfectly direct current; there is no means of directing the flow, either upwards or downwards. Then the window commonly known as the Middlesex Hospital window, was devised, with a view to giving the inflow of air an upward tendency. This window, as many of my hearers know, consists of three horizontal divisions, each of which is hinged on its lower edge, and is made to fall inwards by means of a bar worked by a lever. The defect of this window seems to me to be that all three divisions must perforce be opened at once and that it does not allow of a direct current of air. If, however, these two kinds of windows are combined, and a fall-in or hopper window is placed above a double-hung sash, we get as near an approach to a perfect form of window as possible. The bottom rail of the lower sash should be made much deeper than usual, and instead of the ordinary bead inside a board some five inches deep should be fixed on the sill. With this contrivance if the lower sash be raised some four inches a continuous current of air having an upward direction will be admitted between the upper and the lower sashes. The sides of the hopper light when open must be protected with wings, and the crevice at the bottom where the hinges are must be covered with a wooden slip. Another important point in the prevention of draughts is to see that the air supply to the stove is drawn not from the ward entirely, but from an independent source. This can be done by bringing in a supply of air by a shaft to the back of the fire-place, where it is warmed and passed through suitable gratings into the ward. At the floor level at the back of each bed a small shaft should be made through the wall, and provided with some means of opening and closing, preferably the radiator ventilator made by Ellison, which breaks up the incoming air into four diverging currents. The special function of these openings is to create a movement of air in the most confined space in the ward, under the beds. Shafts for the removal of vitiated air are best placed side by side with the smoke flues, and divided from the latter with thin plates of iron. When fires are not in use an upcast current must be got by means of a gas burner at the foot of the shaft.

Thus far natural ventilation. As a general rule, the system employed in America, where very great attention has been given to the subject, is that of drawing the vitiated air out of the wards by shafts leading into one common aspirating chimney, and of admitting fresh air over steam or hot-water coils placed in a basement entirely devoted to ventilating and warming purposes. Propelling fans are used both for the admission and extraction of air.

In comparing the two systems we are in somewhat of a difficulty, for with the single exception of the Consumption Hospital, at Brompton, there does not to my knowledge exist any hospital in this country entirely dependent on mechanical means for ventilation. One element in the question is that of cost; and though it will be conceded that where the health of patients is concerned cost is a minor consideration, the advantages to be gained must be very real to counterbalance the enormous expense, both initial and of maintenance, involved in such a system as that of the New York Hospital or the John Hopkins Hospital at Baltimore.

Another element for consideration is the multiplication of shafts and the general elaboration of parts upon the perfect order and control of which the success of the whole scheme hangs.

I do not wish for a moment to underrate the vital importance of efficient ventilation in a ward, nor do I desire to cast any doubt upon the success of any particular system. But I think a question that fairly admits of discussion is whether, in view of the comparatively equable nature of our climate, it is not quite possible, with the ordinary means at our disposal, to obtain perfectly satisfactory results.

